# Impact of cannabis use on Schizo-affective disorder

**DOI:** 10.1192/j.eurpsy.2023.1225

**Published:** 2023-07-19

**Authors:** M. Zbidi, W. Bouali, W. Haouari, M. Kacem, S. Khouadja, L. Zarrouk

**Affiliations:** Psychiatry department, Taher sfar University hospital of mahdia tunisia, Mahdia, Tunisia

## Abstract

**Introduction:**

Schizoaffective disorder (SAD) is a nosographic entity characterized by an association of schizophrenic symptoms with thymic episodes. Addictive substance abuse behaviours precede or appear concomitantly with thymic and/or schizophrenic symptoms for the majority of patients.

**Objectives:**

The objective of our work was to specify the sociodemographic ,clinical and therapeutic characteristics of this population and to compare them to a group of schizophrenic patients who do not use cannabis.

**Methods:**

This is a retrospective descriptive study of patients with Schizoaffective Disorder (SAD) meeting the criteria of the Diagnostic and Statistical Manual of Mental Disorders of the American Psychiatric Association, 5th Version (DSM-5), hospitalized between January 2015 and December 2021 in the psychiatry department of the EPS Tahar Sfar Mahdia.

**Results:**

Our sample was composed of two groups: A first group formed by patients with a positive toxicological assessment to tetra-hydro-cannabinol (n=14) and a second group witha negative toxicological assessment (n=36). In SAD subjects using cannabis, the average age at first hospitalization was younger (27.5 years) than in the other groups, hospitalization was earlier (27.27 vs 33.58; p=0.04), the duration in number of days of hospitalization was greater (29.33 vs. 24.67; p=0.02) and they had required during their hospital stay a higher dosage of antipsychotics in equivalent doses of chlorpromazine (723 vs 603; p=0.04). There was a significant difference (p ≤ 0.04) in the psychometric scales (BPRS, SAPS and SANS) in favour of patients who did not use cannabis.

**Conclusions:**

The deleterious psychic effects of chronic cannabis use have long been suspected for a long time. Patients followed for SAD present more frequently than the reference population addictive behaviours towards cannabis which is associated with many negative events affecting clinical symptomatology, evolution, prognosis and therapeutic response.

**Disclosure of Interest:**

None Declared

